# Self-reported energy use behaviour changed significantly during the cost-of-living crisis in winter 2022/23: insights from cross-sectional and longitudinal surveys in Great Britain

**DOI:** 10.1038/s41598-023-48181-7

**Published:** 2023-12-14

**Authors:** Gesche M. Huebner, Clare Hanmer, Ellen Zapata-Webborn, Martin Pullinger, Eoghan James McKenna, Jessica Few, Simon Elam, Tadj Oreszczyn

**Affiliations:** UCL Energy Institute, University College London, Central House, 14 Upper Woburn Place, London, WC1H 0NN UK

**Keywords:** Human behaviour, Energy and society

## Abstract

The winter of 2022/23 has seen large increases in energy prices and in the cost of living in many countries around the world, including Great Britain. Here, we report the results of two surveys, combining cross-sectional and longitudinal analysis, in a sample of about 5400 British households. One survey was conducted early in 2023, the other when participants had signed up to an ongoing research study in the past five years. Thermostat settings were about 1°C lower during the cost-of-living crisis than before, and householders were more likely to turn the heating off when the home was unoccupied. The effort to save energy increased compared to pre-cost-of-living-crisis levels. Using the in-home display more in the cost-of-living crisis than before correlated with greater effort to save energy, supporting the notion that displaying energy data can be a useful tool for energy reductions. Finding it difficult to keep comfortably warm in the home and struggling with meeting heating costs were linked to lower wellbeing, strengthening evidence links between cold, damp, and hard-to-heat homes and negative mental health outcomes. About 40% of respondents lowered the flow temperature of the boiler which might imply that highly tailored information campaigns can be effective in changing behaviour.

## Introduction

The rapid economic recovery following the pandemic had already put strain on the energy markets but the situation developed into ‘a full blown global energy crisis’^1^ following Russia’s invasion into Ukraine in February 2022. Many countries around the world have seen huge increases in energy prices, costs of other goods, and a high inflation. A global analysis showed an increase in energy costs of households by 62.6–112.9% compared to 2021, contributing to a 2.7–4.8% increase in household expenditures^2^. The authors also demonstrated a worsening of global energy poverty, with 2.4–7.9% of the global population moving into energy poverty defined as spending more than 10% of total expenditure on energy. Poorer households tend to have a heavier burden in terms of rates of energy costs in high-income countries such as the UK. Across European countries, European households’ cost of living in 2022 were estimated to increase on average by almost seven percent of total household consumption in 2022^3^.

The turmoil in the UK energy market had already started in 2021 with more than 25 suppliers going out of business or being put in special administration, with the consequence that about 4 million households were switched to new suppliers and often less favourable tariffs^4^.

The cost of living increased dramatically in the UK during 2021 and 2022, with inflation reaching 11.1% in October 2022, the highest value in 41 years^5^. Inflation started to fall slightly subsequently but remained high at 6.8% in July 2023. The high inflation was driven by increases in the costs of consumer good, food items and energy^5^.

The energy price cap, the maximum amount energy suppliers can charge for a unit of energy on a standard tariff, rose by 12% in the UK in October 2021; it increased by a further 54% in April 2022 which was equivalent to a £700 annual increase for typical levels of dual fuel consumption, reaching £1,971 per year^6,7^. It was due to increase by 80% in October 2022 (gas by 91%, electricity by 70%) but the Energy Price Guarantee (EPG) provided a support rate discount to all households with a domestic gas and/or electricity contract, meaning that the typical household bill for a dual-fuel household was about £2500 in Great Britain. Additionally, households received a £400 rebate on their energy bills via the Energy Bills Support Scheme (EBSS) in monthly instalments between October 2022 and March 2023^8^. Those on prepayment meters received vouchers that needed reclaiming with £160 million still to be claimed in April 2023^9^.

The mean temperature of the winter 2022/23 in the UK was 4.3 °C, which is 0.2 °C above the 1991–2020 average but lower than the previous winter. In addition, there were significant cold snaps both in December and January^10^. Government statistics showed that in the fourth quarter of 2022 total final energy consumption was 5.1% lower than in the fourth quarter of 2021. Domestic consumption even reduced by 12% despite the cold December included in this quarter^11^. In the first quarter of 2023 domestic consumption fell by 7.4 per cent compared to the same period in 2021^12^. These reductions in demand likely reflect a response to the high energy prices.

**Figure 1:**
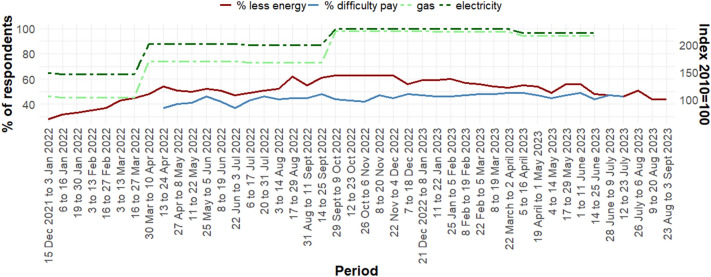
Percentage of respondents finding it at least “somewhat difficult” to afford their energy bills (blue)^14^ and those reporting using less fuel in the home because of increases in the cost of living (red); data based on Great Britain surveys with N ~ 2000 at each data collection timepoint^15^. The dashed lines indicate the real-terms price indices for gas (light green) and electricity (dark green) in the domestic sector compared to 2010 prices^16^.

The cost-of-living crisis can have dramatic impacts on households with wide-ranging implications such as being unable to buy enough food or heating the home to an adequate level^13^. Analysis of the Opinions and Lifestyle Survey (OPN), a fortnightly cross-sectional national survey covering Great Britain with about 2000–2500 respondents at each data collection point showed that 83% of adults reported an increase in their cost of living in March 2022, an increase on the 62% who reported this in November 2021. Price increases for food (90%), gas and electricity bills (79%) and fuel (71%) were cited as the most common reason in winter 2022. Self-reported difficulty in paying energy bills remained high throughout 2022 and 2023 (see Fig. 1).

Given the recency of the cost-of-living crisis not much robust evidence on wider consequences of this period is known, e.g. on health or personal debt. In general, the relationship between economic crises and health is complex and findings depend on level of analysis, the health outcome studied, and the economic indicator used ^17–20^. However, since this crisis in particular is not yet characterized by an increase in unemployment, a commonly-studied indicator, but rather the unaffordability of food and energy, negative health outcomes in the UK are likely, coupled with the already struggling national health system^21,22^. Poor nutrition is associated with negative consequences such as poorer immune status, higher rate of tooth decay, and poorer cognitive function and learning ability^23^. Cold, damp homes have significant physical health effects and increase morbidity and mortality, worsening respiratory and circulatory illnesses in particular^24^. They are linked to excess winter deaths which are higher in the UK than in other countries, with the poorer housing stock likely playing a role^25–27^. Cold, damp homes are also linked to mental health stressors such as persistent worry about debt and affordability, thermal discomfort, and worry about health effects of those living conditions^28,29^.

The price elasticity of energy demand is generally considered as relatively low both in the short and long term, i.e. demand for energy varies comparatively little with changes in price, particularly in the short term. A meta-analysis estimated a price elasticity of all energy demand in the short term of − 0.21, and − 0.61 in the long term, i.e. a 10% increase in energy prices would lead to a reduction in demand of 2.1% to 6.1%. The values for electricity were -0.126 in the short term and -0.365 in the long term; for natural gas − 0.180 and − 0.684, respectively^30^. UK-specific estimates showed a short-term price elasticity of demand for gas from − 0.1 to − 0.28^31^. A more recent study covering the time period from 1975 to 2018 estimated long-term elasticity of electricity demand to -0.607 in the UK^32^; i.e. a 10% rise in the price of electricity would lead to a 6.07% demand reduction. Hence, whilst considered inelastic overall, energy price increases are associated with reduction in demand.

Real-time feedback on energy consumption is associated with significant energy savings^33–35^ though the effect size is estimated to be small in some meta-analyses^36^. The UK government promotes the installation of smart meters and in-home displays (IHD) to ‘help give consumers more control over energy use and spending’^37^ and expects savings for end users through their installation^38^. During the COVID-19 pandemic, a study reported a positive correlation between frequency of the IHD usage and effort to save energy^39^. All participants in the study presented here had a smart meter but increased usage of the associated IHD might be a differentiating factor in greater energy reduction effort.

Energy use per se in homes is largely driven by building factors and only little by attitudinal variables ^40,41^. The impact of psychological variables such as knowledge about climate change and the importance of saving energy on energy saving actions has been researched extensively; however, a large number of psychological variables and outcomes have been used, covering different domains (e.g. water use, heating, recycling, driving), observed behaviour versus self-reported behaviour and behavioural intentions. A 2016 meta-analysis concluded that climate change beliefs only have a small to moderate effect on acting in climate-friendly ways^42^ Also two more recent studies with very large samples found only a small effect of psychological variables on energy behaviours ^43,44^. Given these findings, we decided to not measure any of those psychological variables given their likely small effect, especially during a time when the impact of energy price is presumably dominating strongly. Home retrofitting has been associated with significant energy reductions though often below expectations^45^ but retrofitting measures are not the focus of this study given that they often take substantial time to be implemented and are hence less likely to have been done quickly in response to rapid fuel price increases such as those experienced in 2022. Low-income households are more likely than medium and high-income households to save energy through daily energy savings actions but less likely to invest in retrofit measures, pointing to income as an important variable to consider^46^.

Here, we report the results of a survey study with a sample of about 5,400 respondents (exact number varies slightly by question due to missing or invalid data) collected in early 2023, abbreviated here as *CoL* (Cost-of-Living). Existing participants of the Smart Energy Research Lab project (SERL) all received a postal letter with a survey copy and a link to online completion in February 2023. Response rate was 49%. Owner-occupiers and houses were slightly overrepresented in the sample but household size and income was similar to national averages. Figures S1-S6 in the supplementary material show these and other socio-demographic and building characteristics. Data from the CoL survey were linked with a background survey that participants had filled in when initially signing up to SERL, abbreviated here as *BL* (Baseline) allowing longitudinal analysis for some questions. Both surveys are available online^47^. SERL collects smart meter data plus contextual information^48^.

The main aim of this study was to understand how energy-related behaviours, including the effort to save energy, changed during the cost-of-living crisis, and implications for wellbeing. Specifically, we tested the following hypotheses that were all prespecified^49^ to avoid cherry-picking of results^50^. Compared to the preregistration document, their order has changed to provide a more coherent narrative, and the words “fuel cost crisis” and “this winter” replaced with CoL for greater clarity.

- Hypothesis A: Thermostat settings are lower during the CoL crisis than before the CoL crisis.
- Hypothesis B: Self-reported likelihood to turn heating off when the home is unoccupied is higher during the CoL than before the CoL.
- Hypothesis C:: The effort to save energy is higher during the CoL crisis than before the CoL.
- Hypothesis D: Those who say they use their in-home display more during the CoL crisis report greater effort at saving energy than those who say they use it less.
- Hypothesis E: Those who are doing less well financially report greater effort at saving energy (*a*) and perform energy saving actions more often (*b*).
- Hypothesis F: Those who find it difficult to meet their heating costs have lower wellbeing.
- Hypothesis G: Those who find it difficult to keep comfortably warm have lower wellbeing.

Some additional descriptive information is presented. Any deviations from the preregistration are noted in the text.

### Heating behaviour changes

**Figure 2:**
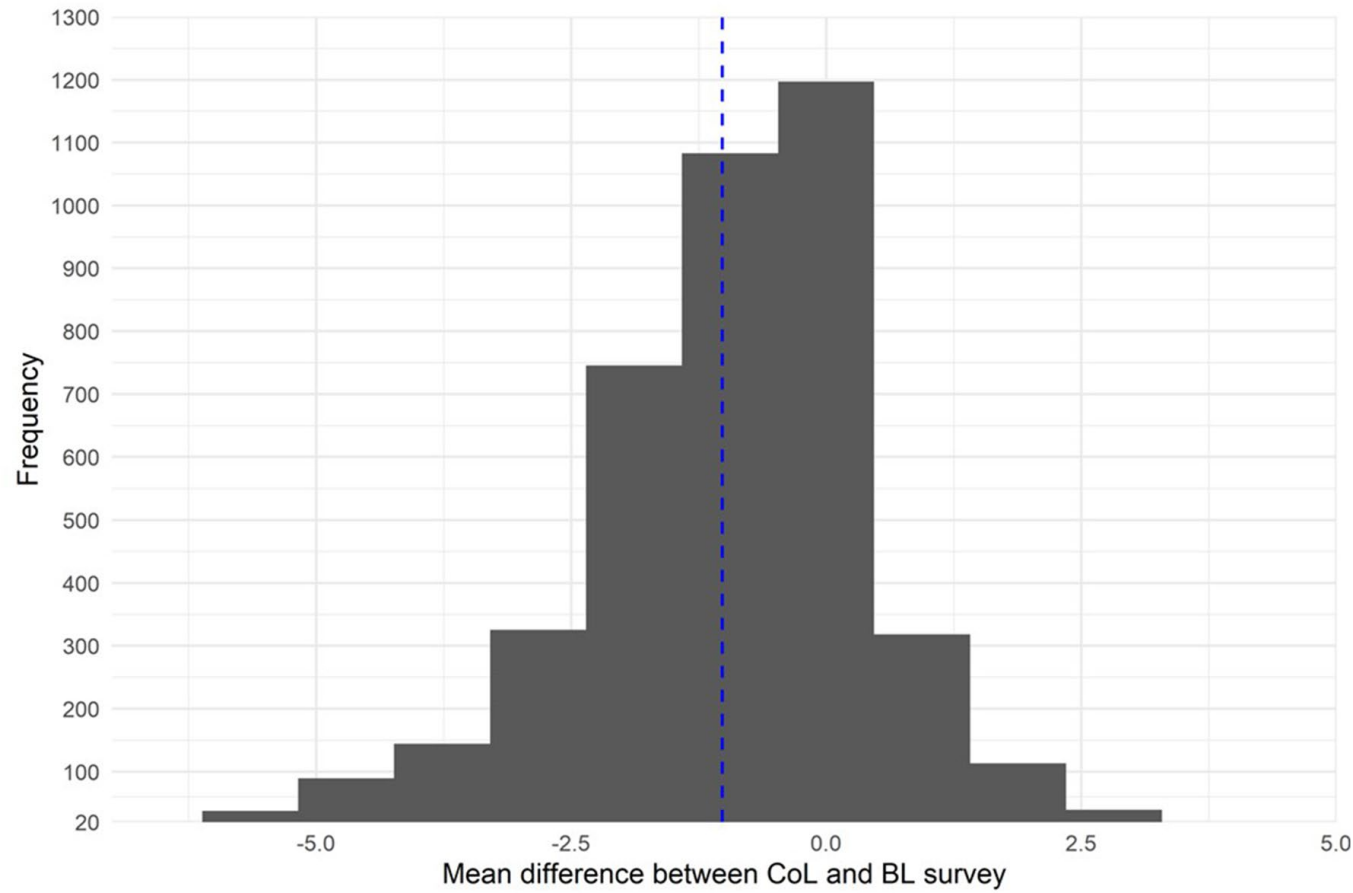
Differences in thermostat settings between CoL and BL survey. Dashed line indicates mean difference. Difference < 0 implies the thermostat temperature was lower during the CoL crisis. N = 4202.

Self-reported thermostat settings during the CoL survey were M = 19.20 °C (SD = 2.15) compared to a baseline mean of M = 20.22 °C (SD = 2.06), i.e. the mean difference was M_diff_ = 1.02 °C (95% CI: 0.96—1.08), see Fig. 2. This difference was highly significant, t(4201) = −32.82, p < 0.001, d = −0.51, confirming Hypothesis A. N = 2491 respondents reported lower thermostat settings during the CoL, with N = 530 respondents reporting an increase, and 1181 reporting no change.

**Figure 3:**
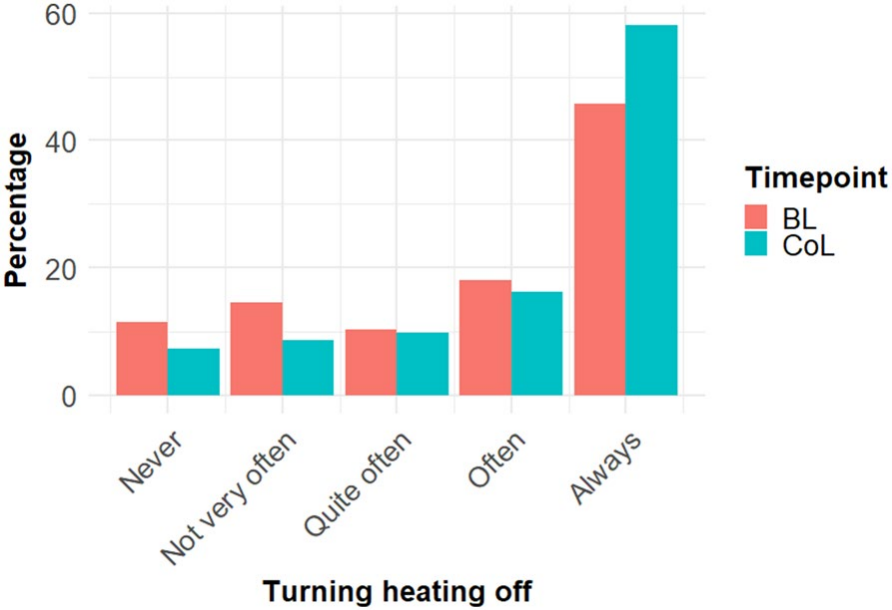
Distribution of answers on frequency of turning heating off at baseline (orange) and during CoL (green). N = 4677.

Participants had been asked at BL and also during the CoL crisis how often they adjusted the heating controls to ensure the heating either was not or was much less likely to come on when their accommodation was unoccupied for more than a day or so. The percentage of those ‘always’ doing it increased from 45.8% (BL) to 58.2% (CoL) (see Fig. 3, N = 4677).

The preregistered repeated measures *t*-test showed that the difference was significant, t(4676) = −54.34, p < 0.001, d = −0.79, with a mean difference of M_diff_ = 1.81 (95% CI: 1.75–1.88). However, given the clear non-normality of the outcome variable, we also performed a non-parametric Wilcoxon rank sum test with continuity correction as a distribution free test, V = 1,114,123, p-value < 0.001, pseudo-median = 2.50 (95% CI: 2.50–2.50). Hypothesis B was confirmed; participants were more likely to turn the heating off this winter than in previous winters.

During the CoL winter 52.1% of participants indicated there were some living spaces, e.g., bedrooms, living or dining rooms, kitchens that they usually did not heat, compared to 35.5% at baseline. An exploratory, non-prespecified Chi^2^ test was used to test for a significant interaction between survey point and yes–no answers, Chi^2^*(1) = 315.99, p < 0.001,N _BL_ = 5658, N_CoL_ = 5641.

Also, 59.4% indicated that they were heating their house for fewer hours than in previous winters (39.3% no, 0.01% do not know, N = 5622).

About 42.4% of respondents indicated that they had reduced the flow temperature of their boiler, i.e. the temperature of hot water supplied to radiators (54.1% no, 3.6% do not know; N = 5278).

In summary, participants reported a significantly reduced use of space heating, including through lowering the boiler flow temperature.

### Wider energy saving actions

**Figure 4:**
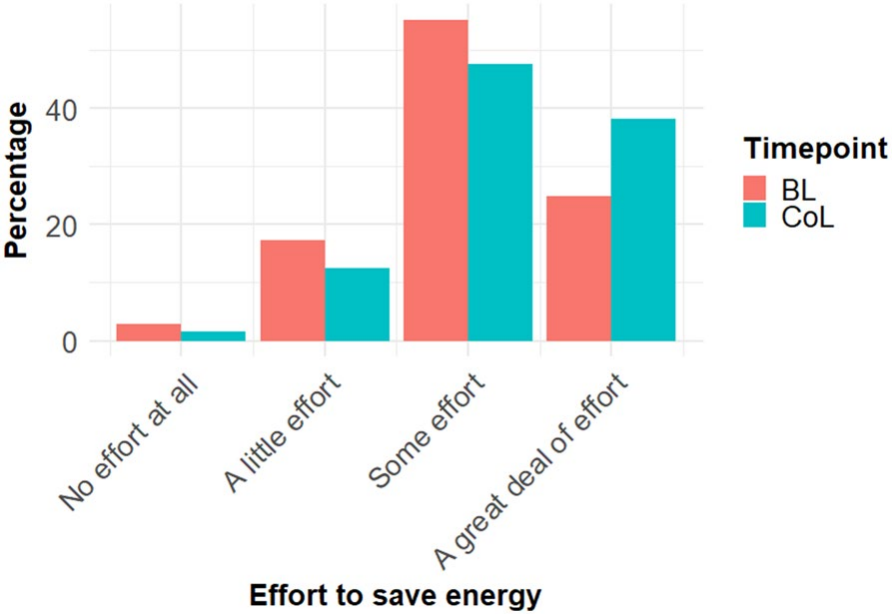
Distribution of answers to the question on effort made to save energy at baseline and during the CoL crisis. N = 5559.

Participants were asked both at BL and during the CoL survey to state how much effort to save energy they made, ranging from “No effort at all” (1) to “A great deal of effort” (4). Figure 4 shows the distribution of responses.

A repeated measures *t-*test showed that effort to save energy was significantly higher during the CoL (M = 3.23, SD = 0.72) than at baseline (M = 3.02, SD = 0.73), t(5558) = 19.30, p < 0.001, d = 0.26, M_diff_ = 0.20 (95% CI: 0.18—0.22). Using a distribution-free test, the Wilcoxon signed rank sum test, confirmed this finding, V = 2,341,185, p-value < 0.001, pseudo median 0.50 (95%CI: 0.50 to 0.50). Hence, Hypothesis C was confirmed.

**Figure 5:**
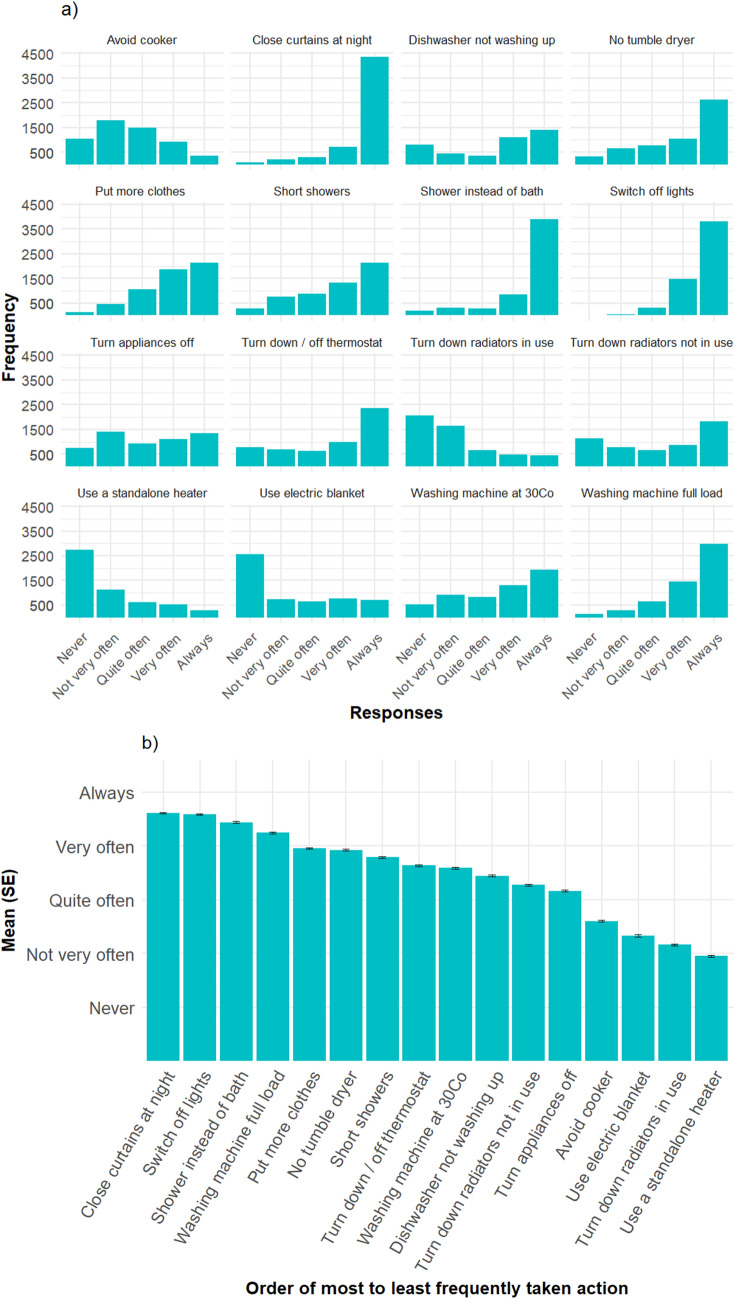
(**a**) Frequencies of responses of how often participants carry out 16 energy saving actions during the CoL. (**b**) Mean values for frequencies of energy saving actions, ordered from most often to least often. Valid N for each action varies between N = 4129 and N = 5699.

In the CoL survey only, frequency of performing energy-saving actions was measured using 16 items covering a range of activities, such as turning off lights, wearing warmer clothes, and reducing cooker usage (see items in question A6 in CoL survey), measured on a scale from 'Never' (1) to 'Always' (5). Figure 5a shows the distribution for each answer and Fig. 5b shows the mean rating of each action ordered from most to least often done.

The actions vary in the frequency with which they are carried out, with closing the curtains, switching off the lights and showering instead of having a bath the most, and using a standalone heater rather than putting the heating on or turning it up, turning down radiators in use, and using an electric blanket, hot water bottle or similar when feeling cold rather than putting the heating on or turning it up the least-often done activities. We calculated Cronbach’s alpha as measure of internal consistency. Using all items, α = 0.68, i.e. below the usual minimum value of 0.7. However, removing the item that had the lowest intercorrelation—using the dishwasher—achieved an acceptable α = 0.71 (95% CI: 0.70–0.72). Hence, we averaged the remaining 15 items for each participant and used it as a measure of frequency of energy saving actions.

**Figure 6:**
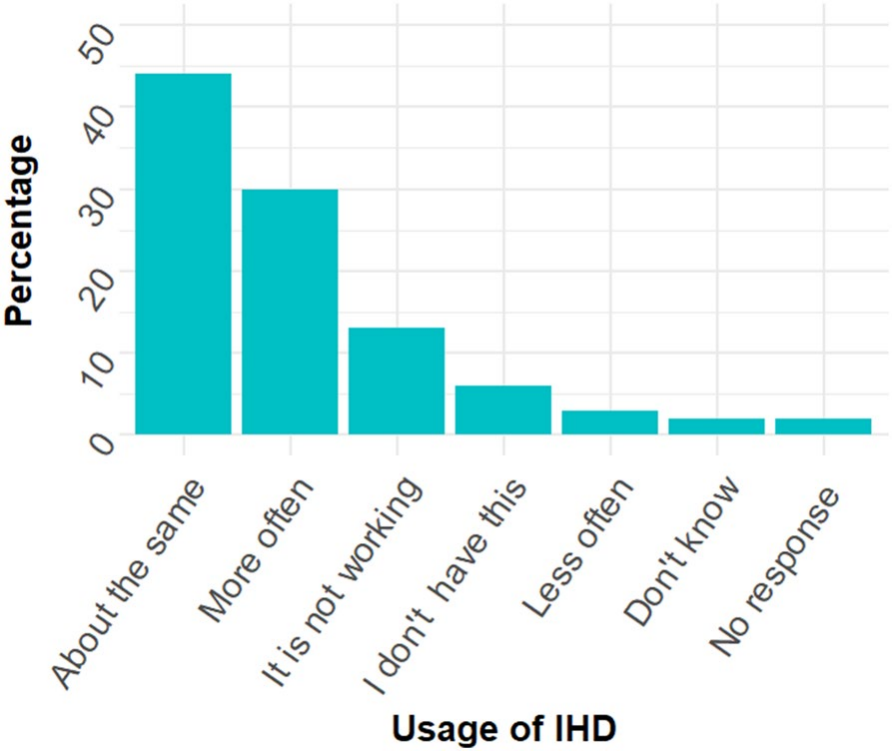
Distribution of responses to the question on usage of the IHD during the CoL compared to the preceding winter. N = 5699.

In the CoL survey, participants were asked how the frequency of their usage of their IHD had changed compared to before the 2022/23 winter. Figure 6 shows the distribution of responses (N = 5699). The most common option chosen was ‘about the same’ (43.9%), followed by ’more often’ (30.5%). Notably, 12.8% of respondents indicated their IHD was not working.

Effort to save energy was M = 3.47 (SD = 0.61) for using the IHD more, M = 3.08 (SD = 0.73) for using it the same, and M = 3.20 (SD = 0.71) for using it less.

A one-way ANOVA with the factor of IHD usage coded as more, less, the same and the outcome of effort to save energy was significant, F(2, 4369) = 167.65, p < 0.001, petasq = 0.07. Pairwise post-hoc comparisons with Bonferroni correction showed that the difference between ‘about the same’ and ‘more often’ was significant, as was the difference between ‘less often’ and ‘more often’ (p < 0.001). Those who used their IHD more this winter made greater effort to save energy than those who used it less, confirming Hypothesis D.

We conducted a regression analysis to understand if financial wellbeing was related to energy-saving actions. The full regression model is presented in Supplementary material Table S1, controlling for tenure, anyone ≤ 16 years in the home, anyone > 85 in the home, anyone not working because of disability, anyone retired, working from home, household size, number of bedrooms, number of bathrooms, building age, and building type as covariates, R^2^ = 0.061 (95% CI: 0.04 to 0.07), F(37, 4540) = 7.90, p < 0.001. Here, we only present the coefficient of financial wellbeing which was β = −0.17 [95% CI: −0.19, −0.15] and was significant at the 0.05 level, i.e. lower financial wellbeing was associated with greater effort to save energy, confirming Hypothesis *Ea*. We repeated the regression using the composite score of frequency of energy-saving actions as outcome, R^2^ = 0.088 (95% CI: 0.07 to 0.10), F(37, 4558) = 11.94, p < 0.001. There was again a negative coefficient of financial wellbeing, β = −0.13 [95% CI: −0.15 to −0.11], p < 0.05, confirming Hypothesis *Eb* (full model Supplementary Material Table S2.)

### Wellbeing implications

Wellbeing was assessed in the CoL survey with two items measuring life satisfaction and finding things in life worthwhile, ranging from 0 (lowest wellbeing) to 10 (highest wellbeing). Life satisfaction is considered as an evaluative approach, asking respondents to make a cognitive assessment of how their life is going overall. The question on things being worthwhile sits in the eudemonic approach^51^. The two items should not be combined into one composite measure^52^, hence, here, separate analyses are carried out for both items. Mean life satisfaction was M = 7.02 (SD = 2.09). The mean value of findings things in life worthwhile was M = 7.37 (SD = 2.08). The median for both measures was Md = 8, the distributions were slightly skewed to the left and overall, wellbeing was relatively high (see Fig. S7).

**Figure 7:**
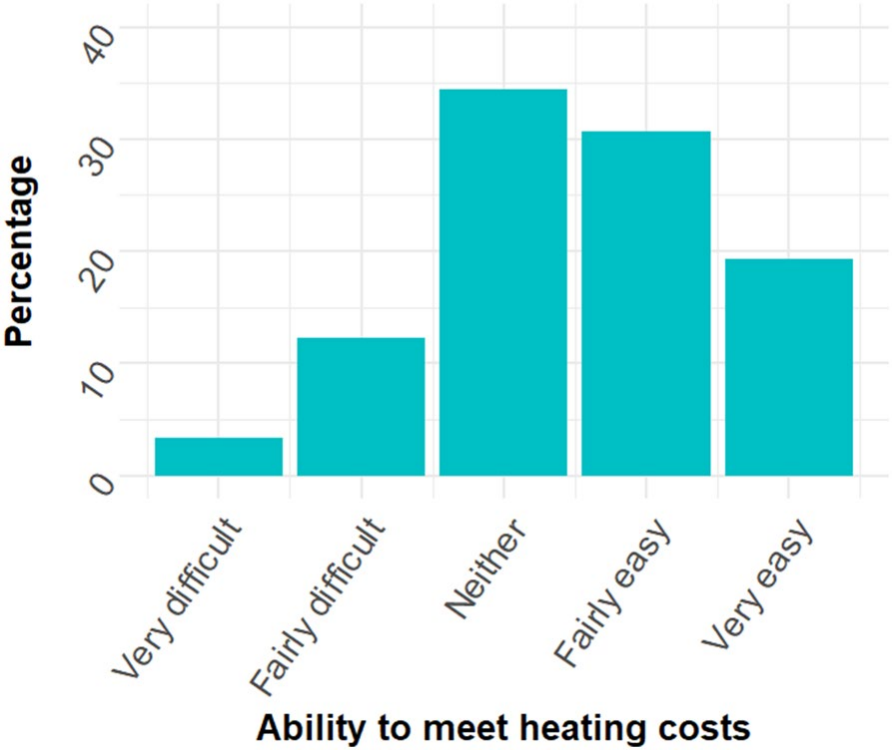
Distribution of responses to the question how easy or difficult respondents find it to meet heating costs during the CoL. N = 5521.

Figure 7 shows the distribution of responses to the questions how easy or difficult it was for households to meet their heating / fuel costs.

There was a significant positive correlation between ease of meeting heating costs and life satisfaction, *r*_s_ (5520) = 0.41 [95% CI: 0.39–0.43], p < 0.001, and things being worthwhile, *r*_s_ (5517) = 0.30 [95% CI: 0.28–0.32], p < 0.001, supporting Hypothesis *Fa* and *Fb*. Those who found it difficult to meet their heating cost had lower wellbeing.

**Figure 8:**
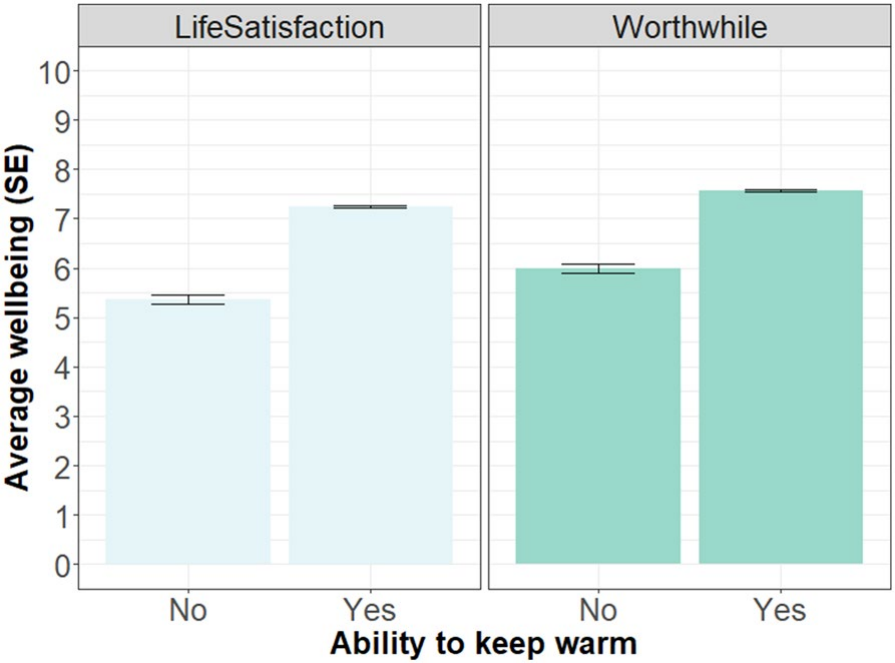
Mean life satisfaction (lighter green) and finding things worthwhile (darker green) split up by being able to keep comfortably warm in the living room or not. Both variables collected in the CoL survey; for life satisfaction N = 5516; for worthwhile, N = 5515.

Across the sample, 12% of respondents said they could not keep comfortably warm, and conversely, 88% could keep warm (with ‘no response’ and ‘do not know’ excluded). An independent samples Welch *t*-test showed that those who struggled to keep warm had significantly lower life satisfaction, t(782.83) = −19.56, p < 0.001, Cohen’s d = 0.87. Those unable to keep warm scored 1.78 points (95% CI: −1.70 to −2.08) lower on life satisfaction than those able to keep warm. They also found things in life significantly less worthwhile, t(777.38) = −15.87, p < 0.001, Cohen’s d = 0.71, with the group unable to keep warm scoring 1.65 points (95% CI: −1.76 to −1.37) lower. See Fig. 8 for the mean life satisfaction and worthwhile values split up by ability to keep warm.

## Discussion and conclusion

The results clearly indicate that substantial behaviour change occurred in British homes during the winter 2022/23 with householders having reduced their thermostat setting, heating fewer spaces and turning the heating off more, and in general making more effort to save energy. All pre-specified hypotheses were confirmed. Given that only households with smart meters were included that currently are found in about 55% of homes and that may differ slightly from other households, there is a limit to generalizability; hence, the following is discussed with the caveat of potentially not being the case for all of Great Britain.

About 40% of respondents reduced the flow temperature of their boiler. This is an action that was heavily promoted in the winter of 2022/23 in public energy awareness campaign^53,54^ whereas it had been little discussed previously. Whilst this study cannot prove it were the information campaigns that created this behaviour change, it is a clear possibility and would support the effectiveness of public information campaigns. Previous research has indicated that public information campaigns do not lead to behaviour change but in those instances, the direct benefits for householders might have been lower compared to the 6–8% gas savings for optimal boiler flow temperature^53,55^. Previous information campaigns were likely also broader and calling for sustained actions whereas changing the boiler flow temperature is one precise one-off action.

Results confirmed the relationship between frequency of IHD usage and energy-saving effort as already reported during the Covid-19 lockdown^39^. Given the correlational nature of the study, this relationship needs to be interpreted with caution; it could indicate that IHDs help with saving energy but also that householders use their IHD to check that their energy saving actions work. In either case, an IHD could be considered a useful tool for householders. Data also showed that over 10% of householders reported their IHD was no longer working. Suppliers, whilst required to provide an IHD at point of smart meter installation, are not responsible for its functionality after the first year^56^. Whilst it is possible that some of those displays indeed do work but consumers do not know how to use them, a different survey also saw significant issues with smart meters displays reported. Hence, a significant and likely increasing number of consumers might not be able to benefit from IHDs and without an IHD smart meters might deliver negligible impact on energy usage^57^. Creating a clear line of responsibility for IHD maintenance and functionality is needed, ideally without putting the burden on consumers.

In terms of specific energy-saving actions taken, results indicate that the most popular energy-reduction actions were not necessarily the most effective ones. The most frequently done behaviour of *closing curtains* can save some energy; however, 87% of UK homes have at least double-glazing where the potential for savings is limited. In addition, the item asked about drawing curtains at night as opposed during the day, limiting when energy saving might occur. The second most common action of *turning off lights* saves comparatively little energy due to the efficiency of modern lighting^58^. Winter energy use is dominated by space heating but of the actions that could reduce demand for space heating only *putting on more clothes* was in the top 5, which included none of the options around turning down thermostats and radiators. This study did not assess which actions householders believe to be most effective but a mismatch between perceived and actual efficiency of energy saving actions has been previously documented^59,60^, highlighting the need to provide specific advice on actions that are supposed to save energy and/or money. Citizen’s Advice, an independent UK consumer organization that provides advice around a range of issues, provided monetary estimates of various energy savings actions though not of all and only covered a selection of actions, not necessarily including all of the most effective ones, e.g. excluding reducing the boiler flow temperature^61^. The Energy Saving Trust, another British energy advice organization, also published advice on quick actions that overlap to a substantial amount with Citizen Advice but also omit actions that e.g. Citizen Advice had as most effective, such as reducing the thermostat setting, but then includes additional ones^62^. Hence, whilst there is substantial evidence available online, it is not an easy task for consumers to understand what actually would be most effective for them, especially since this also heavily depends on factors such as their tenure, their dwelling type but also their personal needs, such as health status.

The study findings also give first evidence that in the cost-of-living crisis winter of 2022/23 lower wellbeing was observed amongst those who struggled to meet their heating costs and struggled to keep warm, consistent with evidence around cold homes and mental health problems^29,63^. Hence, it is likely that the winter of 2022/23 will lead to a wider public health repercussions^64^ though again it needs to be stressed that the work presented in this paper is correlational only.

The substantial behaviour changes observed in this study and reflected in energy reduction in different countries contrast with the smaller energy behaviour changes observed in COVID-19 lockdown. During lockdowns people spent a lot more time at home and large increases in energy use were expected but not observed^39,65^; which could be further indication that energy behaviours are habitual^66–68^—but that despite the low elasticity of energy prices^30^, at a high enough price change, habits break and behaviours change. Zapata-Webborn et al.^69^ have shown that average electricity consumption was 8.4% lower and gas consumption 10.8% in lower than the previous winter in GB homes. Using a dichotomy of low versus high financial wellbeing, the authors showed those with low financial wellbeing made greater energy consumption reductions, strengthening the finding that those who do less well financially make greater effort at saving energy.

To conclude, the cost-of-living crisis has led to substantial changes in energy-use behaviour. Specific information on the most effective energy-saving actions needs to be provided and might be successful in changing behaviour as for changing the flow temperature of boilers. A solution for maintenance of In-Home-Displays should be developed. The cost-of-living crisis winter might have long-lasting consequences on human wellbeing and consequently, medical care costs.

## Methods

### Data collection procedure

This paper reports the result of two survey studies. The main survey was conducted during the cost-of-living crisis (CoL). The survey was sent as a postal letter to 12,001 households on 02/02/2023 with data collection closing on 11/04/2023. This survey was sent as a paper copy but also contained a weblink so that participants were able to respond online. Participants had also filled in a survey when they had signed up to the SERL study initially (BL). Data from both surveys were linked for the present analysis.

### Sample

Response rate for the CoL survey was 49%, with 5827 valid surveys returned. Since not all householders had filled in the BL survey, the linked sample size was N = 5699.

We compared sample characteristics to census data; whilst the comparison is imperfect with the census covering England and Wales and the sample the whole of GB, these data were most comparable in terms of item categories. The sample had an overrepresentation of owner-occupied dwellings of 87% compared to national estimates^70^ of 63% and an underrepresentation of both privately-rented flats with 5% compared to 20% and 7% social housing compared to 17%. Flats were also underrepresented with 12% compared to 22% in the census data and houses overrepresented with 88% compared to 78%.

According to thecensus, 30% of households are occupied by one person and 34% by two people; with the sample having slightly more 2-person households ^71^.

Median household income in the UK before taxes and benefits was £35,000 in the financial year ending in 2022. In the sample, the median income bracket was £30,001—£40,000^72^.

In summary, the sample had an overrepresentation of owner-occupiers and houses but seemed to match household size and income relatively well. Figures S1–S6 in the supplementary material show these and other socio-demographic and building characteristics.

### Variables, statistical models and software

Both surveys are available within the supplementary material. Participants were able to skip questions, resulting in slightly different sample sizes in different analyses. Table 1 summarizes the statistical models and variables used in analysis. Missing data on any variable was an exclusion criteria for the respective analysis.

**Table 1:** Statistical models and variables used for hypotheses testing.

Hypothesis	Statistical test	Variables	Inclusion /exclusion criteria
A. Thermostat settings are lower during the CoL than before the CoL.	Repeated measures t-test, two-sided	Thermostat setting (A1 CoL) Thermostat setting (A5 BS)	In A1 CoL or A5 BS *Do not know* chosen Values below 5 °C and above 30 °C
B. Likelihood to turn heating off when home is unoccupied for a day or more is higher during the CoL than before the Col.	Repeated measures t-test, two-sided	Turning heating off (A10 BS) Turning heating off (A7 CoL)	In A10 BS or A7 CoL *not applicable* or *cannot do this* chosen
C. The effort to save energy is higher during the CoL than before the CoL.s	Repeated measures t-test, two-sided	Energy saving effort (A14 BS) Energy saving effort (A12 CoL)	In A14 BS or A12 CoL *Do not know* chosen
D. Those using their IHD more during the CoL report greater energy saving effort than those using it less.	One-way Anova with posthoc Bonferroni correction	Outcome: Energy saving effort (A12 CoL) Predictors: IHD usage (A11 CoL) coded as *More often*, *less often*, *the same*	In A11 CoL *I do not have this*, *Prefer not to say*, *It is not working* chosen In A12 CoL *Do not know* chosen
E. Those are doing less well financially (a) report greater effort at saving energy and (b) perform energy saving actions more often.	Linear regression	Outcome a: energy saving effort A12 (CoL) Outcome b: mean of frequency of energy saving actions (A6, CoL) Predictors: financial status E1 (CoL) Confounding variables -tenure (B4 BS) -anyone ≤ 16 years in the home (D2 CoL) -anyone > 85 in the home (D2 CoL) -anyone not working bc of disability (D3 CoL) -anyone retired (D3 CoL) -working from home (D4 CoL) -household size (D1 CoL) -number of bedrooms (B6 BS) -number of bathrooms (B1 CoL) -building age (B9 BS) Building type (B1 BS)	In A12 CoL *do not know* chosen. I In A6 *Not applicable, cannot do this* chosen. In E1 CoL *do not know* or *prefer not to say* chosen
F. Those who find it difficult to meet their heating cost have lower wellbeing.	Spearman rank-order correlation	Heating costs (C5 CoL) (a) Life satisfaction (E2 CoL); (b) worthwhile (E3 Col)	In C5 CoL *do not know* chosen
G. Those finding it difficult to keep warm have (a) lower life satisfaction & (b) find things less worthwhile.	Independent samples t-test, two-sided	Outcome: (a) life satisfaction (E2 CoL) and (b) worthwhile (E3 Col) Grouping: keeping warm (C3 CoL) coded as *yes*, *no*	In C3 CoL *do not know* chosen

One additional analysis was done related to the non-heating of spaces, assessed as BL (A11) and CoL (A2) using a Chi^2^ test with no additional exclusion criteria.

The following variables are not used in hypothesis-testing but descriptive statistics are presented:

- During this winter, are there any living spaces (e.g., bedrooms, living / dining rooms, kitchens) in your accommodation that your household does not normally heat? (A2 CoL)
- During this winter, have you reduced the flow temperature of your boiler? (A3 CoL)
- During this winter, are you heating your house for fewer hours than in previous winters? (A4 CoL)

Analysis was performed using RStudio 2022.02.2 R packages: *tidyverse*^73^, *data.table*^74^, *sjPLot*^75^, *apaTables*^76^, *RVAideMemoire*^77^*, RcolorBrewer*^78^*,* and *here*^79^*.*

### Ethics

The study was judged as low risk and approved by the institutional ethics committee within the UCL Institute for Environmental Design and Engineering and assigned the ID “20221215_IEDE_STA_ETH”. All research was carried in accordance with UCL’s Code of Conduct for Research^80^. The study also complied with the general principles of the Declaration of Helsinki^81^.

All participants were non-vulnerable adults with ability to consent. Informed consent was obtained from all subjects: those who returned surveys via post had to include a signed a paper consent form. Those who participated online had to tick a box to indicate consent before proceeding to the survey. Participants were able to skip any question. For statistical disclosure control reasons, we only show data with a frequency of at least ten cases for each value.

### Bias assessment

Risk of bias describes whether the results might have been influenced by flaws in the design, conduct or analysis of a study. This study represents partly a natural experiment in that it attempts to link changes over time to the cost-of-living crisis, and is partly purely observational in nature by taking a cross-sectional snapshot. It is likely that other events happened that impact the answers, such as changes in employment, birth of a child or death of a household member. This could have particularly impacted the longitudinal analysis since the initial data was collected up to five years ago. However, there was no clear expectation on how any of these events might have impacted results.

The sample was biased in the sense that only households with smart-meters were eligible to sign up to the initial study. More than 55% of UK households have smart meters installed^82^. A stratified random sample approach for SERL participant recruitment using region and index of the multiple deprivation (IMD) quintile as criteria and the sample succeeded in being representative for those^48^. It slightly overrepresent owner occupiers and detached dwellings whilst slightly underrepresenting renters and flats; the latter generally having seen fewer smart meter installations. Hence, the results cannot be generalized to the whole of Great Britain, but are here cautiously interpreted as referring to households with smart meters.

To overcome selective reporting, the analysis was prespecified in advance and any deviations from the pre-registration noted in the manuscript.

### Supplementary Information

Supplementary Information 1.

Supplementary Information 2.

Supplementary Information 3.

## Data Availability

All data collected as part of the SERL project are accessible to all accredited UK academic researchers via a Secure Lab environment^83^. The code is likewise available there.
