# Supplementary material for: Self-reported energy use behaviour changed significantly during the cost-of-living crisis in winter 2022/23: insights from cross-sectional and longitudinal surveys in Great Britain
Source: Sci Rep. 2023 Dec 14;13:21683. doi: 10.1038/s41598-023-48181-7 (PMC10721844; doi:10.1038/s41598-023-48181-7)
Supplement: Supplementary file 1 — Supplementary Information 1. [file 41598_2023_48181_MOESM1_ESM.docx]

## Supplementary material – Full regression models

Table S1 presents the full regression analysis using energy saving effort as outcome.

All categories of a variable had at least 25 responses.

Only two variables are significant in the full regression model. Compared to single-person households, larger households make less effort to save energy, though for the largest household size of 5 or more this was not significant. The presence of anyone over 85 years in the households was associated with lower effort at saving energy.

Table S1. OLS Regression results using energy saving effort as outcome.

| Predictor | *b* | *b*  95% CI  *(LL, UL)* | *sr^2^* | | *sr^2^*  95% CI  *(LL, UL)* |
| --- | --- | --- | --- | --- | --- |
| (Intercept) | 3.85** | [3.46, 4.24] |  | |  |
| Financial status | -0.17** | [-0.19, -0.15] | .04 | | [.03, .05] |
| Tenure_Ref: Own / own with mortgage | |  |  | |  |
| Tenure_Rent-free | -0.03 | [-0.33, 0.28] | .00 | | [-.00, .00] |
| Tenure_Rent privately | -0.03 | [-0.14, 0.08] | .00 | | [-.00, .00] |
| Tenure_Shared ownership | 0.01 | [-0.23, 0.26] | .00 | | [-.00, .00] |
| Tenure_Social housing | -0.02 | [-0.12, 0.09] | .00 | | [-.00, .00] |
| BuildingAge_Ref_1900-1929 |  |  |  | |  |
| BuildingAge_1930 to 1949 | -0.00 | [-0.08, 0.08] | .00 | | [-.00, .00] |
| BuildingAge_1950 to 1975 | 0.01 | [-0.06, 0.09] | .00 | | [-.00, .00] |
| BuildingAge_1976 to 1990 | 0.01 | [-0.07, 0.10] | .00 | | [-.00, .00] |
| BuildingAge_1991 to 2002 | -0.01 | [-0.10, 0.08] | .00 | | [-.00, .00] |
| BuildingAge_2003 onwards | -0.02 | [-0.11, 0.08] | .00 | | [-.00, .00] |
| BuildingAge_Before 1900 | 0.02 | [-0.07, 0.10] | .00 | | [-.00, .00] |
| DwellingType_Ref_Converted flats |  |  |  | |  |
| DwellingType_Detached | -0.01 | [-0.18, 0.16] | .00 | | [-.00, .00] |
| DwellingType_Purpose-built flats | -0.04 | [-0.22, 0.14] | .00 | | [-.00, .00] |
| DwellingType_Semi-detached | -0.01 | [-0.18, 0.16] | .00 | | [-.00, .00] |
| DwellingType_Terraced | -0.01 | [-0.18, 0.16] | .00 | | [-.00, .00] |
| HouseholdSize_Ref_1person |  |  |  | |  |
| HouseholdSize_2people | -0.08** | [-0.14, -0.03] | .00 | | [-.00, .00] |
| HouseholdSize_3people | -0.15** | [-0.24, -0.07] | .00 | | [-.00, .01] |
| HouseholdSize_4people | -0.15** | [-0.26, -0.05] | .00 | | [-.00, .00] |
| HouseholdSize_5 or more | -0.12 | [-0.27, 0.02] | .00 | | [-.00, .00] |
| AnyUnder16_Yes | -0.06 | [-0.14, 0.02] | .00 | | [-.00, .00] |
| AnyOver85_Yes | -0.17** | [-0.26, -0.08] | .00 | | [-.00, .01] |
| AnyDisability_Yes | 0.01 | [-0.05, 0.07] | .00 | | [-.00, .00] |
| AnyRetired_Yes | 0.06 | [-0.02, 0.13] | .00 | | [-.00, .00] |
| Working_Ref_Always from home |  |  |  | |  |
| Working_Never from home | 0.01 | [-0.08, 0.09] | .00 | | [-.00, .00] |
| Working_Other | 0.06 | [-0.02, 0.13] | .00 | | [-.00, .00] |
| Working_Sometimes from home | -0.02 | [-0.10, 0.05] | .00 | | [-.00, .00] |
| NoBathroom_NoneSoleUse |  |  |  | |  |
| NoBathrooms_1 | -0.03 | [-0.35, 0.30] | .00 | | [-.00, .00] |
| Bathrooms2 | -0.01 | [-0.33, 0.32] | .00 | | [-.00, .00] |
| Bathrooms3 | -0.01 | [-0.34, 0.33] | .00 | | [-.00, .00] |
| Bathrooms4 or more | 0.13 | [-0.24, 0.50] | .00 | | [-.00, .00] |
| BathroomsNo response | 0.01 | [-0.37, 0.39] | .00 | | [-.00, .00] |
| NoBedrooms_Ref_1 |  |  |  | |  |
| NoBedrooms_2 | 0.08 | [-0.03, 0.19] | .00 | | [-.00, .00] |
| NoBedrooms_3 | 0.10 | [-0.02, 0.22] | .00 | | [-.00, .00] |
| NoBedrooms_4 | 0.10 | [-0.03, 0.23] | .00 | | [-.00, .00] |
| NoBedrooms_5 | 0.13 | [-0.03, 0.28] | .00 | | [-.00, .00] |
| NoBedrooms_6 or more | 0.14 | [-0.10, 0.39] | .00 | | [-.00, .00] |
| NoBedrooms_No response | -0.30 | [-0.69, 0.09] | .00 | | [-.00, .00] |
|  |  |  |  | *R^2^*  = .061** | |
|  |  |  |  | 95% CI[.04,.07] | |
|  |  |  |  | |  |
|  |  |  |  |  |  |

*Note.* A significant *b*-weight indicates the semi-partial correlation is also significant. *b* represents unstandardized regression weights. *sr^2^* represents the semi-partial correlation squared. *LL* and *UL* indicate the lower and upper limits of a confidence interval, respectively.
 * indicates p < .05. ** indicates p < .01.

Table S2 presents the full regression analysis using frequency of energy saving actions as outcome.

Increasing household size compared to a single person household was associated with significantly lower frequency of energy saving actions. A household with at least one retired person performed energy saving actions more frequently.

Table S2. OLS Regression results using frequency of energy saving actions as outcome.

| Predictor | *b* | *b*  95% CI  *(LL, UL)* | *sr^2^* | | *sr^2^*  95% CI  *(LL, UL)* |
| --- | --- | --- | --- | --- | --- |
| (Intercept) | 4.21** | [3.91, 4.51] |  | |  |
| Financial status | -0.13** | [-0.15, -0.11] | .04 | | [.03, .05] |
| Tenure_Ref: Own / own with mortgage | |  |  | |  |
| Tenure_Rent-free | -0.13 | [-0.36, 0.10] | .00 | | [-.00, .00] |
| Tenure_Rent privately | 0.02 | [-0.06, 0.10] | .00 | | [-.00, .00] |
| Tenure_Shared ownership | 0.11 | [-0.07, 0.30] | .00 | | [-.00, .00] |
| Tenure_Social housing | -0.06 | [-0.14, 0.02] | .00 | | [-.00, .00] |
| BuildingAge_Ref_1900-1929 |  |  |  | |  |
| BuildingAge_1930 to 1949 | 0.03 | [-0.03, 0.09] | .00 | | [-.00, .00] |
| BuildingAge_1950 to 1975 | 0.02 | [-0.03, 0.08] | .00 | | [-.00, .00] |
| BuildingAge_1976 to 1990 | 0.01 | [-0.05, 0.07] | .00 | | [-.00, .00] |
| BuildingAge_1991 to 2002 | 0.03 | [-0.04, 0.10] | .00 | | [-.00, .00] |
| BuildingAge_2003 onwards | -0.03 | [-0.10, 0.04] | .00 | | [-.00, .00] |
| BuildingAge_Before 1900 | 0.03 | [-0.03, 0.09] | .00 | | [-.00, .00] |
| DwellingType_Ref_Converted flats |  |  |  | |  |
| DwellingType_Detached | -0.07 | [-0.20, 0.07] | .00 | | [-.00, .00] |
| DwellingType_Purpose-built flats | 0.01 | [-0.12, 0.15] | .00 | | [-.00, .00] |
| DwellingType_Semi-detached | -0.03 | [-0.16, 0.10] | .00 | | [-.00, .00] |
| DwellingType_Terraced | 0.00 | [-0.13, 0.13] | .00 | | [-.00, .00] |
| HouseholdSize_Ref_1person |  |  |  | |  |
| HouseholdSize_2people | -0.14** | [-0.18, -0.10] | .01 | | [.00, .02] |
| HouseholdSize_3people | -0.19** | [-0.25, -0.12] | .01 | | [.00, .01] |
| HouseholdSize_4people | -0.21** | [-0.29, -0.13] | .01 | | [.00, .01] |
| HouseholdSize_5 or more | -0.15* | [-0.26, -0.03] | .00 | | [-.00, .00] |
| AnyUnder16_Yes | -0.01 | [-0.07, 0.05] | .00 | | [-.00, .00] |
| AnyOver85_Yes | -0.05 | [-0.12, 0.02] | .00 | | [-.00, .00] |
| AnyDisability_Yes | 0.02 | [-0.02, 0.06] | .00 | | [-.00, .00] |
| AnyRetired_Yes | 0.07* | [0.01, 0.13] | .00 | | [-.00, .00] |
| Working_Ref_Always from home |  |  |  | |  |
| Working_Never from home | 0.02 | [-0.04, 0.09] | .00 | | [-.00, .00] |
| Working_Other | 0.05 | [-0.00, 0.11] | .00 | | [-.00, .00] |
| Working_Sometimes from home | -0.02 | [-0.08, 0.03] | .00 | | [-.00, .00] |
| NoBathroom_NoneSoleUse |  |  |  | |  |
| NoBathrooms_1 | -0.17 | [-0.42, 0.07] | .00 | | [-.00, .00] |
| Bathrooms2 | -0.16 | [-0.41, 0.09] | .00 | | [-.00, .00] |
| Bathrooms3 | -0.20 | [-0.45, 0.06] | .00 | | [-.00, .00] |
| Bathrooms4 or more | -0.20 | [-0.49, 0.08] | .00 | | [-.00, .00] |
| BathroomsNo response | -0.05 | [-0.33, 0.23] | .00 | | [-.00, .00] |
| NoBedrooms_Ref_1 |  |  |  | |  |
| NoBedrooms_2 | -0.01 | [-0.09, 0.07] | .00 | | [-.00, .00] |
| NoBedrooms_3 | 0.02 | [-0.07, 0.11] | .00 | | [-.00, .00] |
| NoBedrooms_4 | 0.01 | [-0.09, 0.11] | .00 | | [-.00, .00] |
| NoBedrooms_5 | 0.02 | [-0.10, 0.13] | .00 | | [-.00, .00] |
| NoBedrooms_6 or more | 0.03 | [-0.15, 0.22] | .00 | | [-.00, .00] |
| NoBedrooms_No response | -0.02 | [-0.32, 0.28] | .00 | | [-.00, .00] |
|  |  |  |  | *R^2^*  = .088** | |
|  |  |  |  | 95% CI[.07,.10] | |
|  |  |  |  | |  |
|  |  |  |  |  |  |

## Supplementary material – Socio-demographic and building information

The following figures show basic socio-demographic and building information about the respondents of the two surveys. Data collected in the CoL survey are in a green hue; data collected at baseline are in a red hue. Sample size for all figures about ~5500.


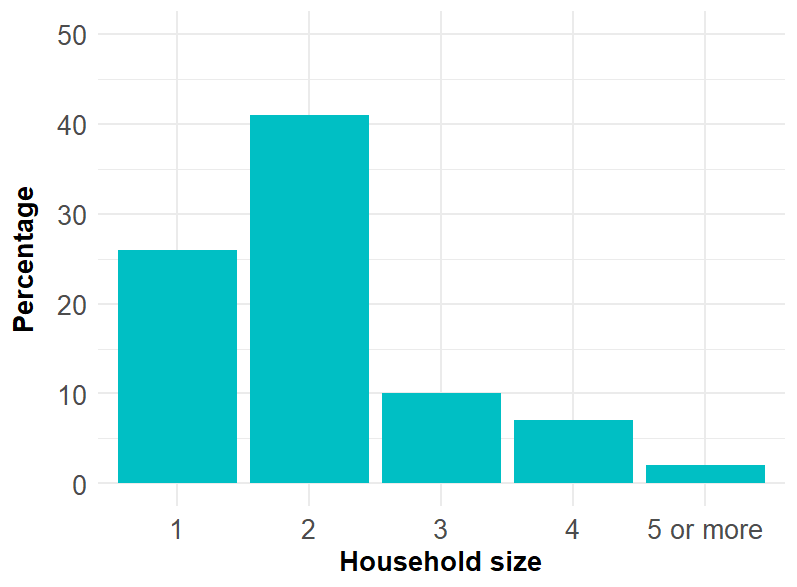


Figure S1. Number of members in the household of the respondents. Collected in the CoL survey.
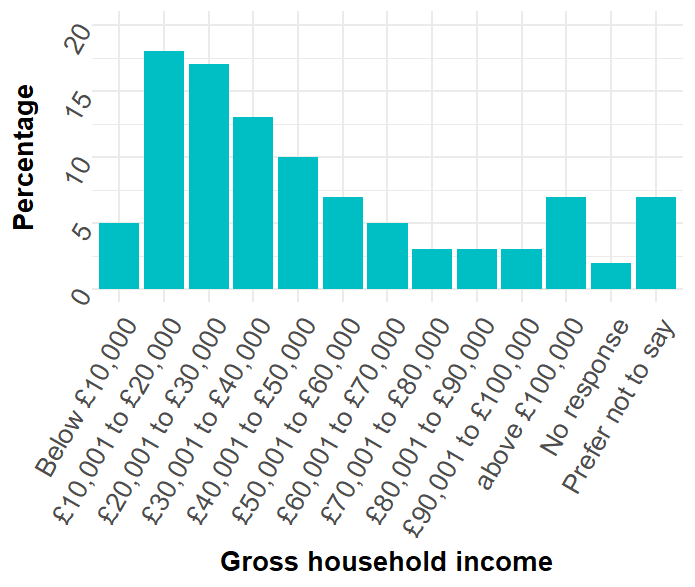


Figure S2. Distribution of gross household income of the respondents. Data collected in the CoL survey.


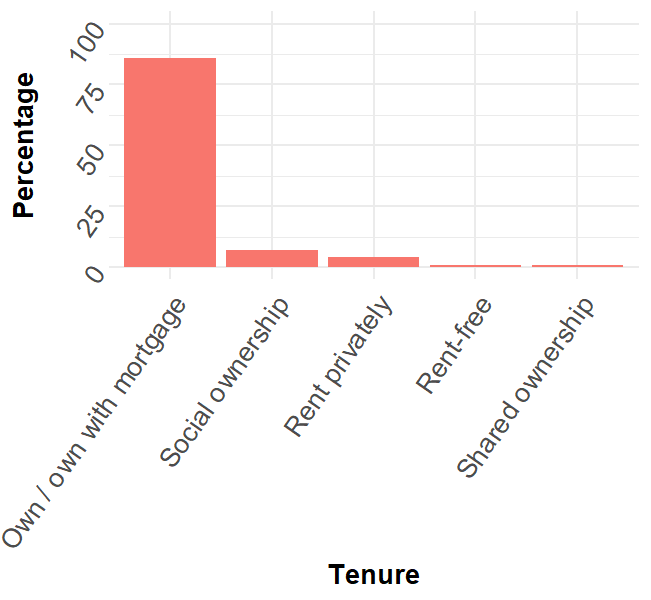


Figure S3. Tenure of the householders who responded to the CoL survey. Data collected at baseline.


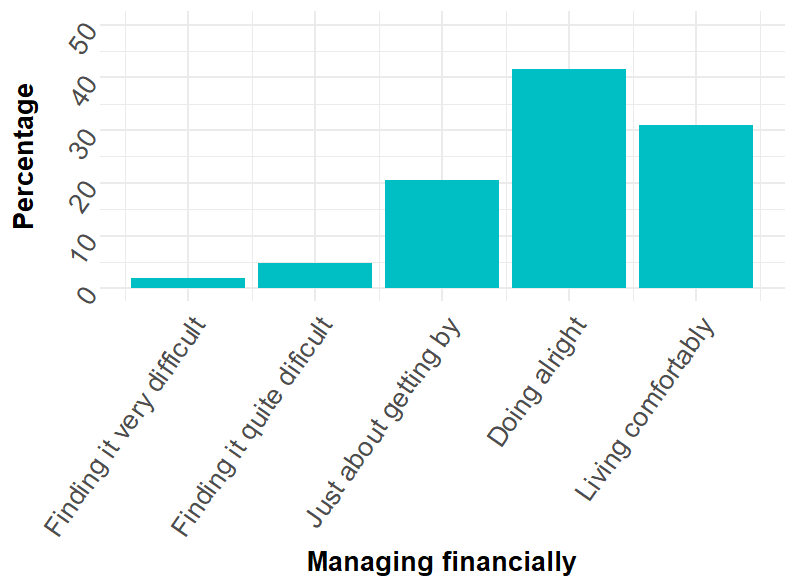


Figure S4. Distribution of responses to the question on financial wellbeing. Collected in the CoL survey.


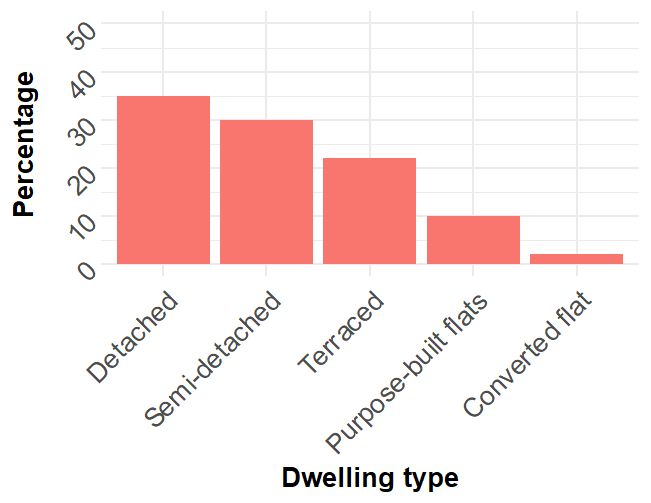


Figure S5. Dwelling type. of the buildings where the respondents lived. Collected at baseline.


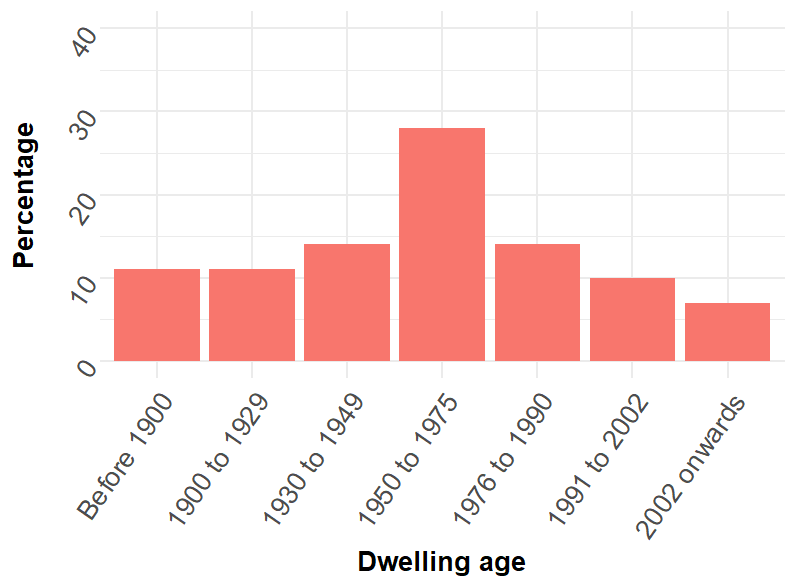


Figure S6. Dwelling age of the buildings where the respondents lived. Collected at baseline.


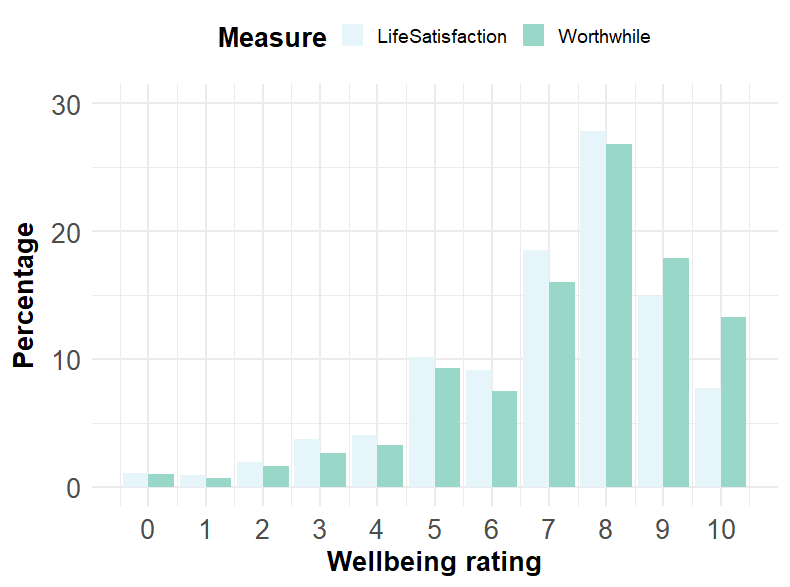


Figure S7. Distribution of responses to the two wellbeing measures life satisfaction (light green) and things in life being worthwhile (dark green), collected in the CoL survey.
