# Supplementary material for: Self-reported energy use behaviour changed significantly during the cost-of-living crisis in winter 2022/23: insights from cross-sectional and longitudinal surveys in Great Britain
Source: Sci Rep. 2023 Dec 14;13:21683. doi: 10.1038/s41598-023-48181-7 (PMC10721844; doi:10.1038/s41598-023-48181-7)
Supplement: Supplementary file 3 — Supplementary Information 3. [file 41598_2023_48181_MOESM3_ESM.docx]

## Supplementary material. TreQ list.

Huebner, G. M., Fell, M. J., & Watson, N. E. (2021). Improving energy research practices: guidance for transparency, reproducibility and quality. *Buildings and Cities*, *2*(1), 1–20. https://doi.org/10.5334/bc.67

| **Tools** | **Check** | **Comments** |
| --- | --- | --- |
| ***Pre-registration*** |  |  |
| This study has pre-analysis plan. | Yes |  |
| *If yes* |  |  |
| URL |  | *https://doi.org/10.17605/OSF.IO/5JDTN* |
| Was it registered before data collection? | Yes |  |
| Does the paper mention and explain deviations from the PAP? | Yes | Yes. Twice a non-parametric test conducted in addition to preregistered test. One exploratory Chi2 test conducted. |
| ***Reporting guidelines*** |  |  |
| This paper follows a reporting guideline. | Yes |  |
| *If yes* |  |  |
| Which one? |  | *STROBE (von Elm, E. et al. The Strengthening the Reporting of Observational Studies in Epidemiology (STROBE) statement: guidelines for reporting observational studies. Lancet 370, 1453–1457 (2007).)* |
| ***Open Data and Code*** |  |  |
| Data/code are publicly available | Yes but only for accredited researchers | The data are part of a project that allows only data access in a secure environment, see https://serl.ac.uk/wp-content/uploads/2021/05/Accessing-SERL-Observatory-data-info-for-researchers-v04a.pdf for details |
| Does the paper make a statement on data and code availability? | Yes, on data and code | All data collected as part of the SERL project are accessible to all accredited UK academic researchers via a Secure Lab environment. The code will likewise be available there (filename: Code_*PaperDoI*_Huebner). |
| *If yes* |  |  |
| What is / are the link(s)? |  | https://serl.ac.uk/wp-content/uploads/2021/05/Accessing-SERL-Observatory-data-info-for-researchers-v04a.pdf |
| Have steps been taken to ensure the data are FAIR? | Yes  No |  |
| Has meta-data been uploaded? | Yes |  |
| ***Preprints*** |  |  |
| Have you uploaded a preprint? | Yes |  |
| *If yes* |  |  |
| What is the link? |  | *https://osf.io/preprints/socarxiv/984yh* |
| *If planned* |  |  |
| Which preprint server/location? |  |  |
